# Medical research: what to expect in a student–supervisor relationship

**DOI:** 10.1186/s12909-022-03851-4

**Published:** 2022-11-10

**Authors:** Alaa Althubaiti, Suha M. Althubaiti

**Affiliations:** 1 College of Medicine, King Saud bin Abdulaziz University for Health Sciences, Mail Code 6656, P.O. Box 9515, Jeddah, 21423 Saudi Arabia; 2 King Abdullah International Medical Research Centre, Jeddah, Saudi Arabia; 3 College of Science and Health Professions, King Saud bin Abdulaziz University for Health Sciences, Riyadh, Saudi Arabia; 4 King Abdullah International Medical Research Centre, Riyadh, Saudi Arabia

**Keywords:** Medical research, Students, Research supervisor, Expectation, Relationship, Role perception

## Abstract

**Background:**

A medical research supervisor is of crucial importance to the undergraduate student enrolled in a research methodology course. A solid relationship between the two is vital to the success of the research project and the overall well-being of the student. The structure of the relationship between a student and a research supervisor is seldom discussed in the context of undergraduate medical research. This study evaluates students’ expectations of their research contributions and their supervisors’ roles.

**Methods:**

This was an observational study in a large health university in Saudi Arabia. A total of 320 medical students enrolled in a two-year medical research program completed an online survey, of a previously validated instrument, that is, Role Perceptions Rating Scale. Demographic questions such as the current level in the research program (junior or senior) were added.

**Results:**

The results showed that most students expected the responsibility to be equally shared between the supervisor and student during the development and execution of the research project. Additionally, students expected the research supervisor to be responsible for the research themes and contents, ensuring access to facilities, and assisting in the actual writing of the final research manuscript. Furthermore, the results indicated differences in expectations between junior and senior students.

**Conclusion:**

This study demonstrates that medical students expect their research supervisors to support them to a significant extent. Understanding medical students’ expectations in a supervisor–student relationship is essential to successful research and collaboration. The evidence gathered in this study has practical implications for educational institutes to base their research training program on these insights. Providing clarity on the expectations and responsibilities of those participating in the research program is crucial, as this would, in turn likely advance the output of the research program and encourage clinicians to join the program as research supervisors.

**Supplementary Information:**

The online version contains supplementary material available at 10.1186/s12909-022-03851-4.

## Background

Research methodology courses are an integral part of the medical curriculum [1]. These courses provide students with the necessary knowledge to formulate a research question, write a research proposal, conduct data collection and analysis, and prepare a research report. Research-oriented educational programs are increasing to incorporate knowledge and improve exposure to supervised research at an early stage [2]. Different aspects of these courses have been evaluated to enhance students’ learning experiences and outcomes [3–8]. One of these aspects is students’ satisfaction with respect to their overall experience [5, 8]. Key determinants of this is the relationship between the student and the research supervisor and the amount of support received from the supervisor [9–11]. Previous results have revealed that these determinants are crucial in improving students’ research experience and outcomes, regardless of students' knowledge level or attitude towards research [8, 12, 13].

Both students and supervisors have expectations of each other [12]. Literature has shown that undergraduate students expect more support in data collection, time management, and theoretical research components (such as in writing the discussion or research objectives) [8, 14]. On the other hand, supervisors expect students to be responsible for completing their research requirements and perceive adequate supervision to be providing direction and fostering a student’s independence in training [15]. A study of undergraduate medical students [16] has identified a range of student concerns, e.g., students feel obliged by their supervisors’ motivations to publish in peer-reviewed academic journals. The priority for publication influences such supervisors to shift from their student’s needs and enhance the student learning experience [16]. Hence, establishing expectations early and reassessing them as needed is considered one of the practices that contributes to a good supervisory relationship [17]. Moreover, regarding those essential to the success of student-supervisor relationships, other determinants have been investigated, such as students’ cognitive skills [18], sex role, level in their research program [19], and their supervisors’ level of expertise or knowledge [20].

Much of the literature on the student-supervisor relationship has focused on a postgraduate research course; few studies [5, 21, 22] have addressed this in an undergraduate research course [15]. Research supervisors in a medical research program are primarily physicians, and although the majority have received adequate research training, [5, 23–25] they experience a few challenges when participating in research, such as finding adequate time for supervision and availability for their students [5, 26, 27]. When implementing a research course in medical curricula, it is important to assess what students want to do in a research project, what they expect from their research supervisors, and how adequately supervisors are able to meet their needs [28, 29]. This will help identify areas of improvement in conducting a research course and possibly enhance the research output of undergraduate medical students.

This study aims to evaluate students’ expectations of their research contributions and their supervisors’ roles using a validated scale. In other words, this study analyses the perceived responsibilities of the supervisors and medical students involved in a research project. The influence of students’ research experiences on their perceived responsibilities is also examined.

We address the following research questions:

- What is the overall satisfaction of medical students with their research supervisors in a medical research program?
- Does satisfaction differ according to academic level in the program?
- What are students’ expectations in terms of their research contribution and their supervisor’s role?
- Finally, do these expectations differ according to academic level in the program and student’s satisfaction with their research supervisors?

## Methods

The present study was observational and was conducted in a large health university in Saudi Arabia. The six-year curriculum design of the college consists of two pre-medical years, two pre-clinical years in which the medical research program is studied, and finally two clinical years. During the research program, students were given a series of research sessions over two years and expected to complete a research project under their research supervisor [5, 6, 30].

### Patient and public involvement

No patients were involved in this study; neither patients nor the public were directly involved in the design, conduct, reporting, or dissemination plans of this research.

### Participants

This study included female and male medical students in their pre-clinical years undergoing their first (juniors) and second (seniors) year in the medical research program. The study used a convenience sampling approach. Those willing to participate were recruited in the study.

### Data collection tool

The Role Perceptions Rating Scale (RPRS) was used to collect data [31]. It is a validated scale that has been used in similar studies [19, 32]. The survey was distributed online using Google Forms.

The scale included 12 items on the topic/course of study, contact/involvement, and thesis. The word thesis was replaced with research manuscript to match the term used for the final assignment in the first and second year of the program, respectively. A few examples of the items include “It is a supervisor’s responsibility to select a promising topic,” “A supervisor should initiate frequent meetings with a student,” and “A supervisor should insist on seeing drafts of every section of the manuscript to review them in a timely fashion.” All items were measured on a 5-point scale, with the minimum and maximum scores ranging from 1 to 5. Higher scores indicate greater agreement on student responsibilities. A score of 3 denotes a neutral response, indicating that the student expected the task to be shared equally. The Cronbach’s alpha reliability coefficient of the RPRS in this study was 0.84 [33]. The validity of the RPRS was ensured through the counsel and evaluation of two experts before application.

The data were collected toward the end of the research program during the 2021–2022 academic year, with three reminders over the course of four weeks. Overall satisfaction with the research supervisor was also measured, and answers were classified as “overall satisfied,” “overall dissatisfied,” and “neither satisfied nor dissatisfied.”

### Data analysis

Statistical analyses were conducted using the JMP 14 software (SAS Institute Inc., Cary, NC, USA). A *p*-value < 0.05 was considered statistically significant. Non-parametric statistical tests were used due to the non-normality of the responses of majority of items based on the Shapiro-Wilk test (*p*-values<0.001). Quantitative variables are reported as mean (standard deviation) or median and range, and categorical variables are expressed as proportions. A Mann–Whitney U test was applied to assess differences in responses between groups and the effect size was reported (with values indicating small effect=0.1, medium effect=0.3, and a large effect=0.5) [34, 35]. A chi-square test was used to evaluate the association between overall satisfaction and students’ level in the medical research program.

## Results

### Overall satisfaction with supervisor’s performance

A total of 320 from an approximate of 500 students enrolled in the medical research program participated in the study. The mean age of participants was 21.5 years (standard deviation = 1.4 years), and there were 170 males (53.1%) and 150 females (46.9%). Most students (64.4%) had an excellent academic grade (grade point average (GPA) = 4.5 – 5). Second-year students in the program accounted for 50.9% of the sample.

Students’ overall satisfaction with the research supervisor was as follows: 146 (45.6%) were satisfied and 104 (32.5%) were dissatisfied. In addition, a total of 70 students (21.9%) stated that they were neither satisfied nor dissatisfied. To facilitate the interpretation of results, students with the level of satisfaction being “neither satisfied nor dissatisfied” were excluded from further analysis [36].

**Fig. 1:**
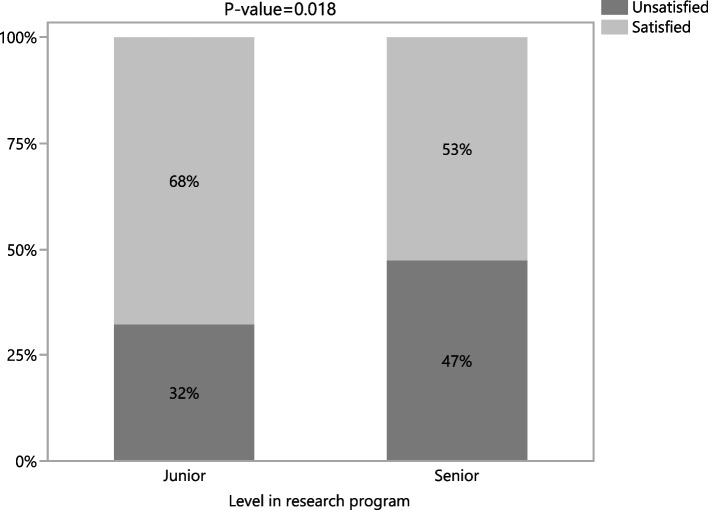
Satisfaction with research supervisor and level in research program

A statistically significant association was found between students’ level in the program and overall satisfaction with the supervisor (chi-square test = 5.56; p-value = 0.018), (Figure 1). Students in the first year of the program (juniors) were significantly more satisfied, compared with those in the second year of the program (seniors) (68% versus 53%).

### Role perceptions rating

Table 1 shows the distribution of students’ responses regarding expectations. The highest agreement between student responses on supervisors’ responsibility was on that supervisors are expected to ensure that students have access to all necessary facilities (*n* = 228, 71.3%). Other items that received high agreement from the students included: maintaining a professional relationship (*n* = 202, 63.1%), initiating frequent meetings (*n*=168, 52.5%), ensuring that the research is finished within the time limit (*n*=166, 51.9%), the direct responsibility of the supervisor toward the methodology of the research (*n*=201, 62.8%), expecting the supervisor’s assistance in the actual writing process (*n*=216, 67.5%), and reviewing the final draft (*n*=223, 69.7%). In addition, the highest disagreement among students was related to the selection of the research topic, where 45.9% stated it is a shared responsibility, 26.6% agreed it is the supervisor’s responsibility, and 18.8% stated it was a student responsibility. Furthermore, the majority (66.6%) of students agreed that the supervisor should support students regardless of their opinion of the students’ capabilities.

**Table 1:** Distribution of students’ answers on different responsibilities in medical research. N(%) are presented

**Item**		**1**	**2**	**Equal responsibility**	**4**	**5**	
1	It is a supervisor’s responsibility to select a promising topic.	4(1.3)	85(26.6)	147(45.9)	60(18.8)	24(7.5)	It is a student’s responsibility to select a promising topic.
2	It is up to the supervisor to decide which theoretical frame of reference is most appropriate.	12(3.8)	154(48.1)	87(27.2)	55(17.2)	12(3.8)	The student has a right to choose theoretical standpoint even if it conflicts with that of the supervisor.
3	A supervisor should direct a student in the development of an appropriate program of research and study.	24(7.5)	64(20)	219(68.4)	5(1.6)	8(2.5)	A student should be able to work out a schedule and research program appropriate to his/her needs.
4	A supervisor should ensure that a student has access to all necessary facilities.	12(3.8)	228(71.3)	57(17.8)	15(4.7)	8(2.5)	Ultimately, the student must find the necessary facilities to complete his/her research.
5	Supervisor-student relationships should be purely professional.	12(3.8)	202(63.1)	36(11.3)	52(16.3)	18(5.6)	Close personal relationships are essential for successful supervision.
6	A supervisor should initiate frequent meetings with a student.	17(5.3)	168(52.5)	119(37.2)	5(1.6)	11(3.4)	A student should initiate meetings.
7	A supervisor should check constantly that a student is on track and working consistently.	119(37.2)	138(43.1)	31(9.7)	20(6.3)	12(3.8)	Students should work independently and not have to account for how they spend their time.
8	A supervisor should terminate the candidate if she/he thinks a student will not succeed.	4(1.3)	27(8.4)	27(8.4)	213(66.6)	56(17.5)	A supervisor should support the student regardless of his/her opinion of the student’s capability.
9	A supervisor should ensure that the research manuscript is finished within the time limit.	34(10.6)	166(51.9)	105(32.8)	3(0.9)	12(3.8)	As long as the student works steadily, she/he can take as long as she/he needs to finish the work.
10	A supervisor has direct responsibility for the methodology and content of the manuscript.	72(22.5)	201(62.8)	43(13.4)	3(0.9)	1(0.3)	A student has total responsibility for ensuring that the methodology and content are appropriate to the discipline.
11	A supervisor should assist in the actual writing of the research manuscript if the student has difficulties and should ensure that the presentation is flawless.	17(5.3)	216(67.5)	15(4.7)	31(9.7)	41(12.8)	A student must take full responsibility for presentation of the research manuscript, including grammar and spelling.
12	A supervisor should insist on seeing drafts of every section of the research manuscript in order to review them in a timely fashion.	49(15.3)	223(69.7)	36(11.3)	1(0.3)	11(3.4)	It is up to a student to ask for constructive criticism from a supervisor.

Table 2 presents a comparison of responses on role responsibility according to the academic level in the research program. Mann–Whitney U test showed statistically significant differences in six of the role perception’s items. The expectation of supervisor’s support during selection of the research topic differed between junior and senior students (z=-9.352; *p*-value <0 .001, large effect size: 0.52). For the selection of the research topic, the median score was 3 for the junior students (range, 2–4), while the senior students had a respective median score of 4 (range, 1–5). Additionally, in comparison to the senior students, junior students were significantly in greater agreement that the selection of the theoretical frame of reference (*p*-value<0.001; effect size: 0.36), ensuring access to facilities (*p*-value<0.001; effect size: 0.31), initiation of meetings (*p*-value = 0.008; effect size: 0.15), ensuring the completion of the research within the time limit (*p*-value < 0.001), and providing feedback and requesting drafts of the manuscript (*p*-value<0.001) are mainly within the supervisor’s responsibility and not a shared responsibility.

**Table 2:** Comparison of responses on role responsibility according to academic level in the research program

Item	Total (*n*=320)		Level in research program		Test Statistic value (z)	Effect size	*P-value
			Junior (*n*=157)	Seniors (*n*=163)			
	Mean	Median (range)^a^	Median (range)	Median (range)			
Selection of topic	3.05	3	3(2-4)	4(1-5)	-9.352	0.52	<0.001
Selection of theoretical frame of reference	2.69	2	2(2-4)	3(1-5)	-6.394	0.36	<0.001
Development of research plan	2.72	3	3(2-5)	3(1-5)	-1.228	0.07	0.22
Access to facilities	2.31	2	2(2-5)	2(1-5)	-5.566	0.31	<0.001
Personal or professional relationship	2.57	2	2(2-5)	2(1-5)	-0.682	0.04	0.49
Meeting initiation	2.45	2	2(2-5)	3(1-5)	-2.636	0.15	0.008
Checking on track	1.96	2	2(1-4)	2(1-5)	-0.704	0.04	0.48
Termination of research candidate	3.90	4	4(2-5)	4(1-5)	-0.417	0.02	0.51
Completion of research within time limit	2.35	2	2(1-4)	3(1-5)	-6.093	0.34	<0.001
Methods and content of research manuscript	1.94	2	2(1-5)	2(1-5)	-1.366	0.08	0.67
Writing the research manuscript	2.57	2	2(2-5)	2(1-5)	-1.283	0.07	0.19
Feedback on research manuscript	2.08	2	2(1-3)	2(1-5)	-5.103	0.29	<0.001

^a^ Minimum=1, maximum=5

^*^The *p*-values were calculated with Mann-Whitney U Test

No statistically significant difference was found in expectations between juniors and senior students in terms of the development of a research plan, preference for professional or personal relationships, checking if the student is on track, possibility to terminate a research candidate, writing methods and content of research manuscript, and assisting in writing manuscript.

We did a subgroup analysis to compare the role perceptions with the overall satisfaction with supervision and determine which items were rated higher by those who were satisfied, and which were rated particularly lower by those who were unsatisfied. Note that a higher rating indicates the perception that the task is a student’s responsibility. Results show that students satisfied with supervisors scored statistically significantly lower compared with unsatisfied students on selection of theorical frame of reference, development of research plan, meeting initiation, termination of research candidate, completion of research within time limit, responsibility for methods and content of research manuscript, and writing/feedback related to research manuscript (all *p*-values<0.001). Therefore, those unsatisfied with their supervisors were less reliant on them. Results are shown in Supplement Table 1.

## Discussion

To the best of our knowledge, this study is among the first to address a gap in the literature by investigating undergraduate medical students’ expectations of their research contributions and their supervisors’ roles. The present study raises interesting questions regarding the role expectations of medical students and provides insights to an improved relationship with research supervisors. A validated scale has been used to determine the expectations. The internal consistency of the scale items has been evaluated in this study, and our results show that these items are internally consistent.

Significant differences in terms of satisfaction with research supervisors were observed among junior and senior students, and we found that satisfaction was higher among junior students. At the start of their research course, students are required to determine their research instrument, in addition to planning their research methodology. Frequent meetings with research supervisors are needed to discuss the different aspects of this research plan. In a previous study on postgraduate and undergraduate students, this stage was found to be the most complex and worrisome in a research course, particularly among undergraduate students [37]. Moreover, our analysis of role perceptions indicated that medical students are highly dependent on their supervisors’ support. Junior students clearly showed greater reliance on their supervisors than seniors. This finding supports the prevailing findings in the literature [38, 39]. Their reliance on their supervisors possibly stems from their lack of understanding concerning the correct methodology for conducting research. However, as students gain a clearer understanding of their research with time, their reliance on their supervisors decreases. Hence, medical research supervisors need to consider a supervisory approach that is adapted to students’ level of research experience [9].

Moreover, our analysis of role perceptions indicated students who were satisfied with their supervisors reported greater reliance on their supervisors than unsatisfied students. Our findings may therefore indicate that supervisors are meeting the expectations of their students and are perhaps readily available to address their questions and concerns.

The increase in the number of undergraduate research programs has created a challenge for educators in terms of providing the best research support and maintaining students’ motivation towards research. Good research is measured by having a clear and important research question and reporting a quality research outcome [40]. Having clear expectations and identifying the roles of each member in a research project are fundamental parts of research collaboration. Previous studies have shown that the quality of postgraduate research supervision depends on the direct influence of the supervisory process and personal traits of students [8, 41, 42]. For appropriate research supervision, characteristics such as the relationship between the quality and style of supervision and the field of study need to be considered [41]. Supervision style must be adjusted in each stage of a research program according to its requirements and student needs [29, 43]. Regarding supervisory practices, supervisors certainly need adequate professional development to be able to use valid, reliable, and effective evaluation systems [42]. Thus, implementing formative and summative evaluation will most likely improve teaching and learning. Furthermore, as limited supervisory training workshops are conducted, [44], more regular faculty enhancement research workshops are needed to support good research practices, particularly for beginning research supervisors [21].

Both supervisors and medical students should be encouraged to discuss research plan and supervision style at the beginning of a research project, rather than focusing only on the selection of a research topic. The role perceptions rating scale should also be completed by each student and can be discussed with the research supervisor. Open dialogue and discussion between student and supervisor are needed. The benefits of this strategy are important in developing the learning environment, enhancing the research experience of both students and supervisors, and promoting student conflict resolution skills. In addition, for students, some of the important practices in a supervisory relationship are asking assertively about what is needed to succeed, properly preparing for supervision meetings, taking full ownership of research projects, playing an active role in managing the supervisory relationship, submitting timely drafts, and keeping their supervisor(s) adequately informed.

### Limitations of the study and future research

The study used a self-report instrument for data collection, which may have introduced response bias [45]. For future studies, gathering data on supervisors’ expectations and matching them with student expectations could be informative. In the present study, collecting the supervisors’ responses was difficult, as most of the focal supervisors have clinical duties in addition to their teaching activities, which affected their response rate. Moreover, since it is vital to explore the needs of students at the start of supervision to ensure effective guidance and support, future research may develop a tool of evaluation for assessing competencies, interests, and needs.

## Conclusion

This study demonstrates that medical students expect greater support from their research supervisors in a range of research activities. These expectations differ as students advance in their research experience, which may imply the important impact supervisors have on beginning students and the further assistance needed by those students. Overall satisfaction with research supervisors was assessed and found to be acceptable. Level of satisfaction was associated with students’ research experience level; junior research students showed a higher level of satisfaction. Instructors and coordinators within a research program should therefore emphasise understanding the relationship between supervisors and students by setting clear roles and clarifying responsibilities and expectations. This in turn will likely advance the output of the research program and encourage clinicians to join the program as research supervisors.

## Supplementary Information

**Additional file 1.**

## Data Availability

The dataset generated and/or analyzed during the current study are not publicly available due to the requirements of the relevant institutional review board. The full dataset and results of statistical analysis following receipt of ethics approval are available from author AA (thubaitia@ksau-hs.edu.sa).
