# Supplementary material for: Medical research: what to expect in a student–supervisor relationship
Source: BMC Med Educ. 2022 Nov 10;22:774. doi: 10.1186/s12909-022-03851-4 (PMC9648019; doi:10.1186/s12909-022-03851-4)
Supplement: Supplementary file 1 — Additional file 1. [file 12909_2022_3851_MOESM1_ESM.doc]

**Supplement Table 1.** Mean, and median (range) for item scores according to satisfaction with research supervisor.

|  | **Satisfaction with research supervisor** | | | | | |  |  |
| --- | --- | --- | --- | --- | --- | --- | --- | --- |
| **Item** | **Unsatisfied**  **(n=104)** | | | **Satisfied**  **(n=146)** | | | **Test Statistic value (z)** | ***P-value** |
|  | **Mean** | **Median** | **Range** | **Mean** | **Median** | **Range** |  |  |
| Selection of topic | 3.16 | 4 | 4 | 3.19 | 3 | 3 | -0.090 | 0.93 |
| Selection of theoretical frame of reference | 3.22 | 3 | 2 | 2.62 | 2 | 4 | 5.653 | <0.001 |
| Development of research plan | 3.01 | 3 | 3 | 2.63 | 3 | 4 | 3.321 | <0.001 |
| Access to facilities | 2.31 | 2 | 3 | 2.36 | 2 | 3 | -1.070 | 0.28 |
| Personal or professional relationship | 2.67 | 2 | 3 | 2.67 | 2 | 3 | -0.347 | 0.72 |
| Meeting initiation | 2.66 | 3 | 3 | 2.29 | 2 | 4 | 3.679 | <0.001 |
| Checking on track | 2.09 | 2 | 4 | 1.85 | 1 | 3 | 1.487 | 0.13 |
| Termination of research candidate | 4.23 | 4 | 4 | 3.74 | 4 | 3 | 6.126 | <0.001 |
| Completion of research within time limit | 3.00 | 3 | 3 | 2.18 | 2 | 2 | 8.865 | <0.001 |
| Methods and content of research manuscript | 2.10 | 2 | 4 | 1.83 | 2 | 3 | 3.382 | <0.001 |
| Writing the research manuscript | 3.27 | 2 | 3 | 2.38 | 2 | 4 | 5.012 | <0.001 |
| Feedback on research manuscript | 2.05 | 2 | 4 | 2.16 | 2 | 2 | -3.935 | <0.001 |
| *The p-values were calculated with Mann-Whitney U Test. Range=minimum-maximum | | | | | | | | |
